# Disability due to knee pain and somatising tendency in Japanese adults

**DOI:** 10.1186/s12891-018-1940-y

**Published:** 2018-01-19

**Authors:** Tomoko Fujii, Hiroyuki Oka, Junji Katsuhira, Juichi Tonosu, Satoshi Kasahara, Sakae Tanaka, Ko Matsudaira

**Affiliations:** 1 Department of Medical Research and Management for Musculoskeletal Pain, 22nd Century Medical & Research Center, Faculty of Medicine, The University of Tokyo, 7-3-1 Hongo, Bunkyo-ku, Tokyo, 113-8655 Japan; 2 Department of Prosthetics & Orthotics and Assistive Technology, Faculty of Medical Technology, Niigata University of Health and Welfare, 1398 Shimami-cho, Kita-ku, Niigata-shi, Niigata, 950-3198 Japan; 3 Department of Orthopaedic Surgery, Kanto Rosai Hospital, 1-1 Kizukisumiyoshi-cho, Nakahara-ku, Kawasaki City, Kanagawa 211-8510 Japan; 4 Department of Pain and Palliative Medicine, Faculty of Medicine, The University of Tokyo, 7-3-1 Hongo, Bunkyo-ku, Tokyo, 113-8655 Japan; 5 Department of Orthopaedic Surgery, Faculty of Medicine, The University of Tokyo, 7-3-1 Hongo, Bunkyo-ku, Tokyo, 113-8655 Japan

**Keywords:** Knee pain, Osteoarthritis of the knee, Somatising tendency

## Abstract

**Background:**

Knee pain is common and related to knee osteoarthritis. However, there is a discrepancy between knee pain and radiographic osteoarthritis. In the general population, knee pain is associated with psychological and cognitive factors, which would be one explanation for the discrepancy. Limited evidence demonstrates that somatization is associated with knee pain. This study examined the association between disability due to knee pain and a high somatising tendency.

**Methods:**

Japanese adults (aged 20–64 years) who had experienced knee pain in the past four weeks were included in this study (*n* = 14,695, 50% women). Data were extracted from a large internet survey. Somatising tendency was assessed using the Somatic Symptom Scale-8 (SSS-8). Disability due to knee pain was categorized into three levels: 1) knee pain without difficulty with activities of daily living (ADL), 2) knee pain with ADL difficulty but without requiring sick leave, and 3) knee pain requiring sick leave. The association between ≥ high somatising tendency (SSS-8 score ≥ 12) as well as very high somatising tendency (SSS-8 score ≥ 16) and disability due to knee pain was examined using logistic regression models adjusted for age, sex, body mass index, depressive symptoms, education level, regular exercise, chronicity of knee pain (≥3 months), osteoarthritis, rheumatoid arthritis, and fibromyalgia.

**Results:**

Greater disability due to knee pain was associated with a higher odds ratio for ≥ high somatising tendency (adjusted odds ratio (aOR) = 2.36 [2.10–2.66] in group 2 vs. group 1, aOR = 3.23 [2.66–3.92] in group 3 vs. group 1). Stronger associations were found for a very high somatising tendency (aOR = 2.80 [2.42–3.23] in group 2 vs. group 1, aOR = 4.51 [3.64–5.58] in group 3 vs. group 1).

**Conclusions:**

Somatization may play a role in disability due to knee pain in the general adult population with knee pain, similar to the role of somatization in low back pain.

## Background

Knee pain is a common symptom. The 12-month prevalence of knee pain was reported to be 29% in people who were 40–79 years old in Nottingham, UK [1]. In Japan, the prevalence of knee pain was reported to be 33% (men 28%, women 35%) in an integrated cohort in which most participants were 60 years of age or older [2]. In a cohort study of Japanese workers who were 19–64 years old, the one-month prevalence of knee pain was 12% [3].

One cause of knee pain is osteoarthritis (OA). However, there is a discrepancy between radiographic knee OA and knee pain; not all people with knee pain have radiographic knee OA, and not all people with knee OA have knee pain [4]. Recently, magnetic resonance imaging (MRI) of the knee has been studied in relation to knee pain [5, 6]. However, even the structural changes assessed via MRI may not always explain knee pain [7]. Felson et al. emphasized the importance of separating the disease process of OA and the syndrome of musculoskeletal pain and disability, which is a common concept in low back pain [8].

Psychological factors predict the incidence, chronicity, and disability of low back pain [9–11]. Similarly, some evidence indicates that psychological factors are also associated with knee pain, which could be one of the explanations for the discrepancy between radiographic changes and knee pain [12, 13]. Somatization, one psychological factor, is “a tendency to experience and communicate somatic distress in response to psychosocial stress and to seek medical help for it” [14]. Somatization often coexists with depression and anxiety [15]. Somatising tendency is a predisposition to be more aware of and to worry about, common somatic symptoms [16]. Longitudinal studies have shown that somatising tendency is not merely a consequence of musculoskeletal pain but a risk factor for multisite musculoskeletal pain [3, 17]. A few studies have reported that somatising tendency was associated with knee pain [3, 18, 19]. However, evidence is limited regarding whether somatising tendency is associated with disability due to knee pain in the general population. This study aimed to examine whether the severity of disability due to knee pain is associated with a high somatising tendency in the general population of adults with knee pain.

## Methods

### Participants

Japanese adults aged 20–64 years with knee pain in the past four weeks (*n* = 14,695) were included in the present study. Data were extracted from a large internet survey of physical and mental health, which was conducted in February 2015. Participants were recruited by an internet research company, United Inc. (Tokyo, Japan), with which more than 1.37 million individuals across Japan have voluntarily registered. The only inclusion criterion for the survey was an age of 20–64 years. Of the approximately 1.25 million eligible individuals, 270,000 individuals were randomly selected and invited by e-mail to complete an online questionnaire. All respondents (*n* = 52,353) provided web-based informed consent and were compensated. Because the focus of this internet survey was not knee pain, the question regarding knee pain, which we created based on the typical question regarding low back pain, was generic. The question with an illustration showing the area of pain asked whether a participant had knee pain persisting for ≥1 day in the past four weeks. The possible responses were as follows: 1) I did not have knee pain (KP0), 2) I had knee pain without difficulty with activities of daily living (ADL) (KP1), 3) I had knee pain with ADL difficulty but without requiring absence from social activities, such as work or school (KP2), and 4) I had knee pain requiring absence from social activities, such as work or school (KP3). Twenty-eight percent of the respondents (*n* = 14,695) answered that they had knee pain (KP1, 2, or 3). Only the individuals with knee pain were included in this analysis. Additionally, respondents were asked whether their knee pain lasted for ≥3 months. The Institutional Review Board of the University of Tokyo approved this study.

### Assessments

#### Somatising tendency

Somatising tendency was assessed using the Japanese version of the Somatic Symptom Scale-8 (SSS-8) [20]. The SSS-8 is a self-administered questionnaire that assesses somatic symptom burden. The SSS-8 was developed as an abbreviated 8-item version of the Patient Health Questionnaire-15 (PHQ-15) to assess the presence and severity of common somatic symptoms [21]. The following somatic symptoms were assessed: 1) stomach or bowel problems; 2) back pain; 3) pain in the arms, legs, or joints; 4) headaches; 5) chest pain or shortness of breath; 6) dizziness; 7) feeling tired or having low energy; and 8) having trouble sleeping. The SSS-8 was used as a reference measure in the Diagnostic and Statistical Manual of Mental Disorders (Fifth Edition) (DSM-5) field trials to facilitate the diagnosis of somatic symptom disorder [22]. The German version of the SSS-8 has good reliability and validity for the general German population [20]. We translated the English version of the SSS-8 into Japanese [23], and the Japanese version of the SSS-8 has been linguistically and psychometrically validated [24]. The total SSS-8 score was categorized, as in Gierk et al., into the following five groups: no to minimal (0–3); low (4–7); medium (8–11); high (12–15); and very high (16–32) [20].

#### Covariates

Demographic information was also collected in the survey. Body mass index (BMI) was calculated based on self-reported body weight and height as weight (kg)/height (m)^2^. BMI was categorized into 3 levels (< 25, 25–29, and ≥30). The participants were asked whether they engaged in exercise regularly over the past year, such as walking and jogging, that lasted for ≥30 min. The possible responses were none, 1–2 times per month, once per week, or more than twice per week. Engaging in regular exercise was defined as exercising more than twice per week. We also collected self-reported information on the chronic conditions for which the respondents were seeking treatment, including OA, rheumatoid arthritis (RA), and fibromyalgia.

Depressive symptoms were assessed using the Patient Health Questionnaire-2 (PHQ-2), an assessment composed of two questions from the Patient Health Questionnaire-9 [25]. The Japanese version of the Patient Health Questionnaire was previously validated [26]. The PHQ-2 assesses whether an individual has experienced depression and anhedonia within the past two weeks. Although each item is rated on a scale of 0–3 in the original PHQ-2, the present study used the National Center of Neurology and Psychiatry version of the Japanese PHQ-2, which gives each item a binary response of yes or no (1/0) [26]. Therefore, the possible scores for the PHQ-2 were 0, 1, or 2. Individuals who answer yes to at least one question are suspected of experiencing depression, and closer assessment of these individuals is recommended.

### Statistical analysis

Initially, the demographic data were examined using descriptive statistics. The characteristics of the participants were compared according to three knee pain groups using the chi-square test. Whether the severity of disability due to knee pain was associated with ≥ high somatising tendency (SSS-8 score ≥ 12) and very high somatising tendency (SSS-8 score ≥ 16) was examined using logistic regression models. The outcome variables were 1) ≥ high and 2) very high somatising tendency, and they were analyzed in separate regression models. The independent variable was disability due to knee pain (3 levels). The participants who had knee pain without any ADL difficulty (KP1) were treated as the reference group. The chronicity of knee pain was also examined as an independent variable. The final multiple models were adjusted for age (5 levels), sex, BMI (3 levels), PHQ-2 (3 levels), education level (any college or no college), regular exercise (yes/no), and whether the participant was seeking treatment for OA, RA, or fibromyalgia. These potential confounders, which would be associated with both somatising tendency and knee pain and/or were adjusted for in previous studies, were chosen a priori based on the literature [12, 16, 27, 28]. Multicollinearity was not suspected as all variance inflation factors (VIFs) were < 2. The odds ratios (ORs) and their 95% confidence intervals (CIs) were estimated. Analyses were conducted using SAS version 9.4 (SAS Institute, Inc., Cary, NC, USA). All analyses were two-sided, and an α-level of 0.05 was considered statistically significant.

## Results

Participant characteristics are shown in Table 1. The mean age (± standard deviation) was 45.6 ± 11.9 years, and half of the participants (49.5%) were women. Among the participants with knee pain, most (82.8%, *n* = 12,161) had knee pain without any ADL difficulty (KP1), 13.3% (*n* = 1954) had knee pain with ADL difficulty but without requiring sick leave (KP2), and only 4.0% (*n* = 580) reported using sick leave due to knee pain (KP3). Individuals in the greater disability group were more likely to be men, unmarried, and lack a college education. The proportion of participants with a PHQ-2 score of 2 was higher in the greater disability groups, and the proportion of participants with OA, RA, and fibromyalgia was also higher in the greater disability groups. The proportion of participants with a high or very high somatising tendency was higher in the greater disability groups.

**Table 1:** Characteristics of the participants with knee pain

	All (*n* = 14,695)	Knee pain without difficulty in ADL (*n* = 12,161)	Knee pain with difficultly but without sick leave (*n* = 1954)	Knee pain with sick leave (*n* = 580)	p-value*
Age (%)					< 0.001
20–29	1942 (13.2)	1569 (12.9)	271 (13.9)	102 (17.6)	
30–39	2903 (19.8)	2345 (19.3)	411 (21.0)	147 (25.3)	
40–49	3462 (23.6)	2875 (23.6)	441 (22.6)	146 (25.2)	
50–59	4322 (29.4)	3599 (29.6)	588 (30.1)	135 (23.3)	
60–64	2066 (14.1)	1773 (14.6)	243 (12.4)	50 (8.6)	
Sex (%)					0.0003
Men	7421 (50.5)	6082 (50.0)	1000 (51.2)	339 (58.5)	
Women	7274 (49.5)	6079 (50.0)	954 (48.8)	241 (41.6)	
BMI (%)					0.0003
< 25	11,078 (75.4)	9228 (75.9)	1431 (73.2)	419 (72.2)	
25–29	2811 (19.1)	2311 (19.0)	381 (19.5)	119 (20.5)	
≥ 30	806 (5.5)	622 (5.1)	142 (7.3)	42 (7.2)	
Marital status (%)					< 0.001
Not married	6249 (42.5)	5069 (41.7)	884 (45.2)	296 (51)	
Married	8446 (57.5)	7092 (58.3)	1070 (54.8)	284 (49)	
Education (%)					0.003
No college	7820 (53.2)	6402 (52.6)	1077 (55.1)	341 (58.8)	
Some college	6875 (46.8)	5759 (47.4)	877 (44.9)	239 (41.2)	
Exercise (%)					0.016
No regular exercise	11,585 (78.8)	9577 (78.8)	1524 (78)	484 (83.5)	
Regular exercise	3110 (21.2)	2584 (21.3)	430 (22)	96 (16.6)	
PHQ-2 (%)^a^					< 0.001
0	9415 (64.1)	8028 (66.0)	1060 (54.3)	327 (56.4)	
1	2546 (17.3)	2054 (16.9)	399 (20.4)	93 (16.0)	
2	2734 (18.6)	2079 (17.1)	495 (25.3)	160 (27.6)	
Chronic knee pain (%)					< 0.001
No	4906 (33.4)	4096 (33.7)	509 (26.1)	301 (51.9)	
Yes	9789 (66.6)	8065 (66.3)	1445 (74.0)	279 (48.1)	
Osteoarthritis (%)	607 (4.1)	328 (2.7)	209 (10.7)	70 (12.1)	< 0.001
Rheumatoid arthritis (%)	229 (1.6)	138 (1.1)	67 (3.4)	24 (4.1)	< 0.001
Fibromyalgia (%)	81 (0.6)	38 (0.3)	27 (1.4)	16 (2.8)	< 0.001
SSS-8 (%)					< 0.001
No to minimal	5262 (35.8)	4679 (38.5)	418 (21.4)	165 (28.5)	
Low	4175 (28.4)	3542 (29.1)	526 (26.9)	107 (18.5)	
Medium	2510 (17.1)	2069 (17.0)	363 (18.6)	78 (13.5)	
High	1434 (9.8)	1079 (8.9)	284 (14.5)	71 (12.2)	
Very high	1314 (8.9)	792 (6.5)	363 (18.6)	159 (27.4)	

*ADL* activities of daily living, *BMI* body mass index, *PHQ-2* Patient Health Questionnaire-2, *SSS-8* Somatic Symptom Scale-8

*p-value from the chi-square test

^a^PHQ-2 items are commonly scored 0 to 3, but the items in the Japanese PHQ-2 were scored yes/no (0/1)

The results of the final logistic regression model are shown in Table 2. Disability due to knee pain was significantly associated both with ≥ high and very high somatising tendency after adjusting for age, sex, BMI, PHQ-2, education, regular exercise, OA, RA, fibromyalgia, and the chronicity of knee pain. The participants who had knee pain with ADL difficulty were more than twice as likely to have at least a high somatising tendency compared to those who had knee pain without difficulty (adjusted OR = 2.36 [2.10–2.66], *p* < 0.001). Individuals who had knee pain requiring sick leave were more than three times as likely to have at least a high somatising tendency compared to those who had knee pain without ADL difficulty (adjusted OR = 3.23 [2.66–3.92], *p* < 0.001). The results for very high somatising tendency were similar, but stronger associations were found. The odds ratios for very high somatising tendency were 2.80 (95% CI [2.42–3.23], *p* < 0.001) in the participants who had knee pain with ADL difficulty and 4.51 (95% CI [3.64–5.58], *p* < 0.001) in the individuals who had knee pain requiring sick leave compared with those who had knee pain without ADL difficulty. Chronicity of knee pain was significantly associated with ≥ high somatising tendency (OR = 1.28 [1.16, 1.41], *p* < 0.001) but not with very high somatising tendency (OR = 1.12 [0.98, 1.28], *p* = 0.103).

**Table 2:** Associations between high (SSS-8 ≥ 12) or very high somatising tendency (SSS-8 ≥ 16) and disability due to knee pain from multiple logistic regression models (*n* = 14,695)

	SSS8 ≥ 12			SSS8 ≥ 16		
	OR [95% CI]^a^	*p-value*	Type 3 *p-value*	OR [95% CI]^a^	*p-value*	Type 3 *p-value*
Disability due to pain			< 0.001			< 0.001
Knee pain without difficulty in ADL	1			1		
Knee pain with difficulty but without sick leave	2.36 [2.10, 2.66]	< 0.001		2.80 [2.42, 3.23]	< 0.001	
Knee pain with sick leave	3.23 [2.66, 3.92]	< 0.001		4.51 [3.64, 5.58]	< 0.001	
Chronic knee pain yes vs. no	1.28 [1.16, 1.41]	< 0.001		1.12 [0.98, 1.28]	0.103	

*SSS-8* Somatic Symptom Scale-8, *OR* odds ratio, *CI* confidence interval, *ADL* activities of daily living

^a^Adjusted for age, sex, body mass index, depressive symptoms (Patient Health Questionnaire-2 (PHQ-2)), education, regular exercise, osteoarthritis, rheumatoid arthritis, and fibromyalgia

## Discussion

This study is the first to report a significant association between disability due to knee pain and high somatising tendency in Japanese adults. Individuals who had knee pain with difficulty in ADL were more than twice as likely to have a very high somatising tendency compared with those who had knee pain without difficulty in ADL, and those who had knee pain requiring sick leave were more than four times as likely to have a high somatising tendency.

Knee pain is a common symptom, one cause of which is knee OA. However, a discrepancy between knee pain and radiographic knee OA has been observed [4]. A systematic review reported that this discordance can be partly explained by the fact that radiographic knee OA is often defined only based on anterior-posterior and/or lateral views, without a skyline view, which would miss knee OA in the patella-femoral joint [29]. The review also reported that the discordance varied according to the knee pain definition. However, the authors concluded that there was still discordance between knee pain and radiographic knee OA. Therefore, one approach involves separating knee pain and its related disability from the disease process of OA [8], which is a concept similar to that used for low back pain. Knee pain and knee OA share some risk factors, such as older age, being overweight, and having previous knee injuries [28, 30]. In addition, psychological factors, such as depression and somatising tendency, seem to play some role in knee pain, which could be one explanation for the discrepancy between radiographic changes and knee pain [12, 13]. Assessing these psychological factors may help predict the onset and prognosis of knee pain in general and occupational health practices.

The results of the current study add to those of the few previous studies that found an association between somatising tendency and knee pain. As part of a large international survey of musculoskeletal symptoms (the Cultural and Psychosocial Influences on Disability [CUPID] study), Matsudaira et al. used a self-administered questionnaire in Japanese to examine pain in six anatomical regions [3]. Somatising tendency was assessed using a subset of items from the Brief Symptom Inventory [31]. The odds of experiencing any knee pain or disabling knee pain in the past month was approximately three times higher in the participants who had ≥2 distressing somatic symptoms compared to those who had no such somatic symptoms. In the CUPID study in Sri Lanka, a similar but weaker association was found, with a prevalence ratio of 1.8 [19]. These two studies were cross-sectional reports, and the causal direction was unknown. Palmer et al. followed working-aged subjects for 18 months in a postal survey in the UK [18]. A higher somatising tendency was associated with new onset of knee pain and its persistence. The present study further suggests the possibility that a high somatising tendency may lead to greater disability and sick leave utilization in people with knee pain.

The factors associated with disability in knee OA patients have been reported in the literature. Older age, female sex, lower social class, obesity, lower quadriceps strength, comorbid health problems, helplessness, and knee pain severity (rather than radiographic severity) were associated with disability or worsening of physical function in people with knee OA [32–36]. Reduced work productivity is typically assessed in terms of absenteeism (days off from work) and presentieesm (productivity loss while at work). A study on reduced work productivity due to knee problems found that a low 12-Item Short Form Health Survey (SF-12) Physical Component Summary score, semi-manual labor or manual labor, and a high maximum knee pain score were associated with presenteeism in people with chronic knee pain and joint space narrowing [37]. A low SF-12 Mental Component Summary score was associated with absenteeism in this study, although reduced work productivity was primarily attributed to presenteeism. For low back pain, the risk factors for chronicity/disability have been extensively studied and are reflected in the guidelines for the management of low back pain [38]. Psychological distress, depressive mood, somatization, work-related factors, pain severity, functional impact, and prior episodes of low back pain have all been reported as prognostic factors [38]. In a recent prospective study, baseline disability, age, and somatization were independently associated with follow-up disability in patients with chronic low back pain [39]. Our results suggest that the factors associated with disability due to knee pain in the general population may not be the same as those in knee OA patients. Somatising tendency may play some role in disability due to knee pain, which is similar to low back pain. When there is a large discrepancy between radiographic changes and knee pain-related disability, it may be useful for clinicians to assess the patient’s somatising tendency and consider a potential psychological approach.

It is plausible that individuals with a high somatising tendency are more aware of and worry about their knee symptoms and consequently avoid activities that may exacerbate their symptoms [40]. Avoidance behavior may result in disuse and disability. In a previous study, somatising tendency was associated with knee pain especially when the pain was chronic or disabling, although the study was cross-sectional [41]. Therefore, somatising tendency is speculated to be more important for disability related to knee pain rather than the occurrence of knee pain. However, due to the cross-sectional design of the current study, this conclusion cannot be determined. Future longitudinal studies are needed.

Chronicity of knee pain was associated with ≥ high somatising tendency but not with a very high somatising tendency. A prospective study found an association between baseline somatising tendency and knee pain persistence [18]. Chronic pain should lead to greater concern regarding the symptom. Determining a conclusion from our results is difficult. People often experience knee pain intermittently. We inquired only about knee pain occurring within the past four weeks. Therefore, it is possible that a participant who did not have chronic knee pain in the past four weeks experienced chronic knee pain previously. Prospective studies are needed to examine the association between somatising tendency and the course of disability due to knee pain.

The strength of the present study is its large sample size with information on relevant covariables. The participants were not recruited in clinical settings; therefore, the possibility of bias due to seeking medical treatment should be low. However, some limitations should be noted. Due to the cross-sectional study design, the causal direction of the association between disability due to knee pain and somatising tendency is unknown. In the survey participants who did not have knee pain and were not included in this study (*n* = 37,658), the proportions of subjects in the SSS-8 categories of no to minimal, low, medium, high, and very high were 63.8%, 21.4%, 8.6%, 3.4%, and 2.8% respectively, and the proportions of these subjects with high and very high somatising tendencies were much lower than those of the subjects with knee pain. Because the SSS-8 questions assess joint pain, SSS-8 scores should be higher in people with knee pain than those in people without knee pain. In addition, we suspected that chronicity of knee pain was an important covariate that should be examined with disability due to knee pain simultaneously in the regression models. Therefore, we included only the participants with knee pain. Individuals with more disabling knee pain would have had higher SSS-8 scores than those who had knee pain without ADL difficulty. However, the definition of a very high somatising tendency was a high SSS-8 score (≥16). Therefore, we believe that the very high somatising tendency in these participants would not be solely attributed to knee pain. Because the focus of our internet survey was not knee pain, standard questionnaires regarding knee pain and function, such as the Western Ontario and McMaster Universities Osteoarthritis Index (WOMAC), were not used [42]. The questions and responses regarding knee pain and disability in the survey were generic to reduce the burden on the survey participants. Usually, PHQ-2 items are scored 0–3, but the Japanese version of the PHQ-2 used in our study has only binary responses. Although this questionnaire is recommended as a screening tool to assess the necessity of further evaluation for depression by the National Center of Neurology and Psychiatry, misclassification is possible, which would be non-differential. Finally, the participants in the present study were recruited online; therefore, our results may not be generalizable to the general Japanese population.

## Conclusion

High somatising tendency was associated with greater disability due to knee pain in adults. Assessing and addressing somatising tendency may help predict patient outcomes and facilitate the treatment of patients with knee pain in general clinical practice.

## Abbreviations

ADL: Activities of daily living
BMI: Body mass index
CI: Confidence interval
CUPID study: Cultural and Psychosocial Influences on Disability study
DSM-5: Diagnostic and Statistical Manual of Mental Disorders (Fifth Edition)
MRI: Magnetic resonance imaging
OA: Osteoarthritis
OR: Odds ratio
PHQ-15: Patient Health Questionnaire-15
PHQ-2: Patient Health Questionnaire-2
RA: Rheumatoid arthritis
SF-12: 12-Item Short Form Health Survey
SSS-8: Somatic Symptom Scale-8
VIFs: Variance inflation factors
WOMAC: Western Ontario and McMaster Universities Osteoarthritis Index
